# Glucagon-like peptide-1 and the exenatide analogue AC3174 improve cardiac function, cardiac remodeling, and survival in rats with chronic heart failure

**DOI:** 10.1186/1475-2840-9-76

**Published:** 2010-11-16

**Authors:** Que Liu, Christen Anderson, Anatoly Broyde, Clara Polizzi, Rayne Fernandez, Alain Baron, David G Parkes

**Affiliations:** 1 Amylin Pharmaceuticals Inc., San Diego, CA, USA 92121; 2 Isis Pharmaceuticals, Carlsbad CA, USA; 3 Arena Pharmaceuticals, San Diego CA, USA

## Abstract

**Background:**

Accumulating evidence suggests glucagon-like peptide-1 (GLP-1) exerts cardioprotective effects in animal models of myocardial infarction (MI). We hypothesized that chronic treatment with GLP-1 or the exenatide analog AC3174 would improve cardiac function, cardiac remodeling, insulin sensitivity, and exercise capacity (EC) in rats with MI-induced chronic heart failure (CHF) caused by coronary artery ligation.

**Methods:**

Two weeks post-MI, male Sprague-Dawley rats were treated with GLP-1 (2.5 or 25 pmol/kg/min), AC3174 (1.7 or 5 pmol/kg/min) or vehicle via subcutaneous infusion for 11 weeks. Cardiac function and morphology were assessed by echocardiography during treatment. Metabolic, hemodynamic, exercise-capacity, and body composition measurements were made at study end.

**Results:**

Compared with vehicle-treated rats with CHF, GLP-1 or AC3174 significantly improved cardiac function, including left ventricular (LV) ejection fraction, and end diastolic pressure. Cardiac dimensions also improved as evidenced by reduced LV end diastolic and systolic volumes and reduced left atrial volume. Vehicle-treated CHF rats exhibited fasting hyperglycemia and hyperinsulinemia. In contrast, GLP-1 or AC3174 normalized fasting plasma insulin and glucose levels. GLP-1 or AC3174 also significantly reduced body fat and fluid mass and improved exercise capacity and respiratory efficiency. Four of 16 vehicle control CHF rats died during the study compared with 1 of 44 rats treated with GLP-1 or AC3174. The cellular mechanism by which GLP-1 or AC3174 exert cardioprotective effects appears unrelated to changes in GLUT1 or GLUT4 translocation or expression.

**Conclusions:**

Chronic treatment with either GLP-1 or AC3174 showed promising cardioprotective effects in a rat model of CHF. Hence, GLP-1 receptor agonists may represent a novel approach for the treatment of patients with CHF or cardiovascular disease associated with type 2 diabetes.

## Introduction

Glucagon-like peptide-1 (7-36) (GLP-1) is an endogenous incretin hormone that modulates insulin-mediated effects on glucose uptake and metabolism [1-3]. GLP-1 receptors are found in the heart, and several lines of evidence suggest GLP-1 may have cardioprotective benefits [4]. Therapeutic use of GLP-1 is limited by its rapid degradation by dipeptidyl peptidase-4 (DPP-4). Exenatide, a synthetic version of the 39-amino acid peptide exendin-4 not susceptible to cleavage by DPP-4, was originally isolated from the salivary secretions of the Gila monster lizard and shares several glucoregulatory properties with GLP-1 [5,6]. AC3174 ([Leu^14^]exendin-4) is an analog of exenatide with a single amino acid substitution that has similar glucoregulatory properties to both GLP-1 and exenatide [7]. Accumulating evidence from both animal and human studies suggests GLP-1 receptor agonists can improve insulin sensitivity and activate c-AMP mediated signaling pathways in cardiac muscle cells [8-11].

Several studies have demonstrated a strong association of whole-body insulin resistance with chronic heart failure (CHF) [12,13], suggesting an important role of insulin resistance and/or altered glucose homeostasis in the pathophysiology of CHF. Since the failing heart utilizes glucose rather than free fatty acids as an energy source [14,15], treatment with GLP-1 or exenatide may improve both cardiac glucose metabolism and cardiac function in CHF [16]. Additionally, acute treatment with GLP-1 or exenatide has shown cardioprotective effects in several animal models of ischemia and perfusion injury [16-20], and recent data has reported that exenatide significantly reduces intimal hyperplasia in insulin resistant animals independent of exenatide-associated weight loss [21]. Further, in pilot studies continuous infusion of GLP-1 improved cardiac function in patients with myocardial infarction (MI), improved left ventricular (LV) function in patients with CHF, and was beneficial in patients with type 2 diabetes with CHF [22-24]. However, no response was observed with acute GLP-1 infusion in patients with established cardiac disease [25].

The purpose of the present study was to determine whether chronic treatment with GLP-1 or the exenatide analog AC3174 has cardioprotective effects in a rat model of MI-induced CHF, to identify specific aspects of cardiac and metabolic function affected by GLP-1 or AC3174, and to evaluate some potential mechanisms for any observed effects.

## Materials and methods

### Induction of myocardial infarction

All experiments were performed in accordance with the protocols and guidelines approved by the Institutional Animal Care Committee and the NIH guide for the Care and Use of Laboratory Animals. MI was induced in male Sprague-Dawley rats (200-225 g) by the supplier (Charles River Laboratories, Wilmington, MA) using a previously described procedure [26]. Briefly, the left anterior descending coronary artery was ligated with a silk suture after an incision in the fourth intercostal space under anesthesia (2% Isoflurane). The same surgical procedure was also performed on a group of rats (sham-operated) except that the suture around the coronary artery was not ligated. The wound was then closed with metal clips, and the rats were allowed to recover for one week before being shipped. Two weeks after MI, rats with an LV infarct size between 20% and 45%, as estimated by echocardiograph at the authors' facility, based on LV chamber kinetic movement [27]. These rats were then randomly assigned to treatment groups and alzet pumps were implanted. Randomized rats were infused subcutaneously with 2.5 pmol/kg/min GLP-1 (GLPL, n = 11), 25 pmol/kg/min GLP-1 (GLPH, n = 12), 1.7 pmol/kg/min AC3174 (3174L, n = 13), 5 pmol/kg/min AC3174 (3174H, n = 7), or vehicle (25% DMSO + 75% H_2_O, control n = 13-16, sham n = 10) via osmotic Alzet pumps (Durect Corp, Cupertino, CA) for 11 weeks. AC3174 and GLP-1 doses were selected to provide equivalent plasma exposure [28]. Echocardiography was performed at 0, 1, 5, 7, and 11 weeks of treatment (2, 3, 7, 9, and 13 weeks post-MI). At 10 weeks of treatment (12 weeks post-MI), MI rats were subjected to a treadmill test. During the last week of treatment, blood samples for fasting plasma insulin and glucose levels were collected before terminal hemodynamic measurements were recorded.

### Echocardiography

Echocardiograms were recorded under light anesthesia (50 mg/kg ketamine hydrochloride plus 10 mg/kg xylazine intraperitoneally) using a Hewlett-Packard 5500 system equipped with S12 (5-12 MHz) phased-array and L15 (7-15 MHz) linear transducers. Transthoracic Doppler echocardiography was performed as previously described [29]. Briefly, short-axis images were obtained at the papillary muscle level and 2 D guided M-mode tracings were recorded at a speed of 150 mm/s. Anterior and posterior end-diastolic wall thickness and LV internal dimensions were measured and % fractional shortening (FS%) was calculated. Left atrial (LA) volume, LV end-diastolic volume (LVEDV), LV end-systolic volume (LVESV), LV ejection fraction (LVEF), LV end-diastolic dimension (LVEDD), and LV end-systolic dimension (LVESD) were measured and calculated from apical views [30,31].

Pulsed-wave Doppler spectra of mitral inflow were obtained from the apical 4-chamber view. The peak velocities of early (E) and late (A) filling waves, and E wave deceleration rate were measured. LV diastolic filling was assessed by E/A ratio. Compliance was assessed by E wave deceleration rate. Aortic velocity time integral (VTI) and LV outflow track diameter were determined, then stroke volume (SV) and cardiac output (CO) were calculated according to the formula: CO = Aortic VTI × [π(LV outflow diameter/2)^2^] × heart rate [32]. Images were digitally acquired and stored for offline analysis by a trained sonographer blinded to the study groups.

### Exercise performance

Immediately before the treadmill test at 12 weeks post-MI (10 weeks of treatment), baseline plasma lactate levels were measured via tail vein puncture in conscious rats fasted for 5 hours. At the time of the treadmill test, 2 rats were simultaneously placed on a 2-track treadmill (Columbus Instruments, Columbus, OH) at a constant 5% grade enclosed by a metabolic chamber (Oxymax Deluxe, Columbus Instruments) through which airflow was maintained at a constant flow rate. Basal measurements were recorded over a period of 8 to 10 minutes. The treadmill was then started at a velocity of 8 m/min for 3 minutes, followed by 12 m/min for 3 minutes, and then maintained at 18 m/min until rats reached exhaustion. The endpoint for the treadmill test was determined by a rat's inability to maintain the pace of the treadmill and remain on the electric shock grid for over 6 seconds. Exercise capacity (EC) was calculated as EC (kgm) = body weight (kg) × degree of grade × running distance. Oxygen consumption (VO_2_) was measured as described [32]. Plasma lactate was again measured one minute after the treadmill test.

### Hemodynamics

Hemodynamic measurements were obtained under anesthesia (2% isofluorane) at 13 weeks post-MI (11 weeks of treatment). A 2F micromanometer-tipped transducer (Millar Instruments, Houston, TX) connected to a PowerLab (8/30) system (ADInstruments, Colorado Springs, CO) was inserted into the right carotid artery to record systolic and diastolic blood pressure. The pressure transducer was then advanced into the LV to measure LV systolic (LVSP) and end-diastolic (LVEDP) pressures, the first derivative of LV pressure over time (± dp/dt) and heart rate (HR).

### Biochemical determinations

Plasma glucose and insulin levels were monitored after an overnight fast at 13 weeks post-MI. Blood samples (20 μl) were collected from a tail vein. Glucose and insulin were measured using an Elite glucometer (Bay, Elkhart, Indiana) and Ultrasensitive rat insulin ELISA kit (Crystal Chem Inc, Chicago, Illinois), respectively. Baseline fasting plasma insulin and glucose were utilized to calculate the homeostasis model assessment (HOMA) of insulin sensitivity. The estimate of insulin resistance from the HOMA score was calculated as described by Matthews et al [33].

### Body composition analysis and histopathological examination

Body weights of conscious rats were monitored weekly. After completion of hemodynamic measurements, rats were deeply anesthetized with 5% isoflurane. Lean and fat mass were measured by quantitative magnetic resonance (QMR) (EchoMRI; Echo Medical System, Huston, TX) as described by Tinsley et al [34]. After QMR measurement, rats were sacrificed under anesthesia and hearts were excised. The atria were trimmed from the ventricles, then the right ventricle, left ventricle, and lung were separated and weighed. LV tissues were immersion-fixed in 10% buffered formalin. Each heart was cut in cross section at four levels from apex to base and prepared for routine histological analysis. The infarct portion of the LV was measured as previously described [35].

### Immunoblot analysis

Cardiac ventricular tissue of post-MI CHF rats from control, GLPH and 3174H treated groups were homogenized in lysis buffer and homogenized tissue extracts were run on Bio Criterion XT Precast Gels and transferred to PVDF membranes (all from BioRad, Hercules, CA). The membrane was immunoblotted with antibodies directed against AKT2, cardiac sarcoplasmic reticulum Ca^2+ ^ATPase (SERCA2), eNOS, glucose transporters (GLUT 1 and GLUT 4), GAPDH, or PI3-kinase-beta, and an HRP conjugate for the secondary antibody (all antibodies from Abcam, Cambridge, MA). Membranes were developed using SuperSignal West Dura Substrate (Pierce, Rockford, IL), and assessed on an Alpha Imager using chemifluorescence. AlphaEase FC software version 1.4.0 was used for quantification. Results were obtained in integrated density value units per 10 μg total protein.

Purified plasma membranes (PM) and cytosolic (Cyt) fractions were prepared using density gradient centrifugation as previously described [36]. Translocation of GLUT-1 and GLUT-4 was analyzed by assessing the protein contents in the PM preparations expressed as percentage of total protein expression in the PM and Cyt fractions [37].

### Statistics

Two-way repeated measures ANOVA followed by Bonferroni multiple comparison tests were used to test group differences in LV function and remodeling over time. When a significant interaction was found (p <0.05), differences between groups were analyzed for each time point. Treadmill test data, body composition, western blot, and hemodynamic measurements were analyzed by one-way ANOVA followed by Bonferroni multiple comparison or nonparametric Kruskal-Wallis test followed by Dunnett's multiple comparison. Within-group comparisons of baseline and Peak VO_2_, lactate and glucose data were analyzed by Student's paired *t *test. Time to death due to CHF was evaluated using Kaplan-Meier survival curves. A log-rank trend test for both GLP-1 and AC3174 was performed to evaluate dose-response trend between control, low, and high doses. The trend analysis was performed using equally-spaced weights with no adjustment made for multiple comparisons. Results are shown as mean ± SEM.

## Results

### General characteristics of rats with CHF

**Figure 1:**
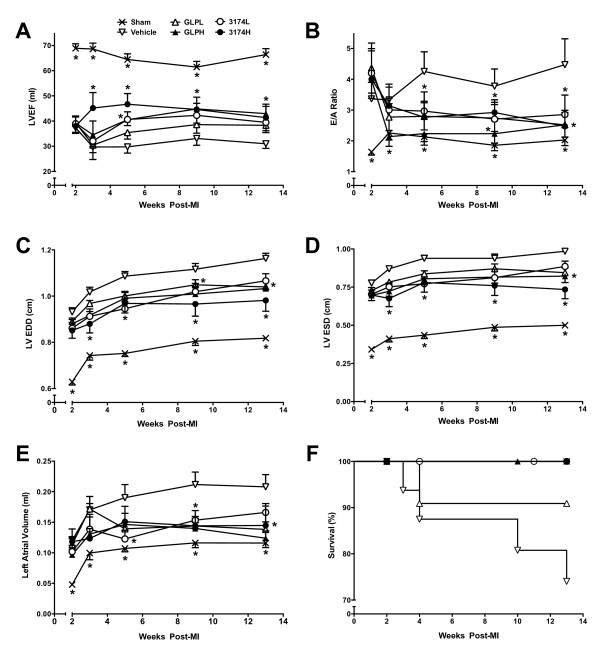
**Effect of chronic treatment with GLP-1 or AC3174 on cardiac function and survival**. (A) Left ventricular ejection fraction (LVEF). At weeks 3 and 5 post-MI, 3174H LVEF was significantly less than GLPL LVEF (p < 0.05). At week 3 post-MI, 3174H LVEF was significantly less than 3174L LVEF (p < 0.05). (B) Doppler ratio for early (E) to late (A) transmitral flow velocity (E/A ratio). (C) Left ventricular end-diastolic dimension (LVEDD). (D) Left ventricular end-systolic dimension (LVESD). (E) Left atrial volume. (F) Kaplan-Meier survival curves. The number of chronic heart failure (CHF) related deaths (from 2 weeks post-MI) and the total number in each group were sham 0/10; control 4/16; GLPL 1/12; GLPH 0/12; 3174L 0/13 and 3174H 0/7. *p < 0.05 versus vehicle-treated control group.

**Figure 2:**
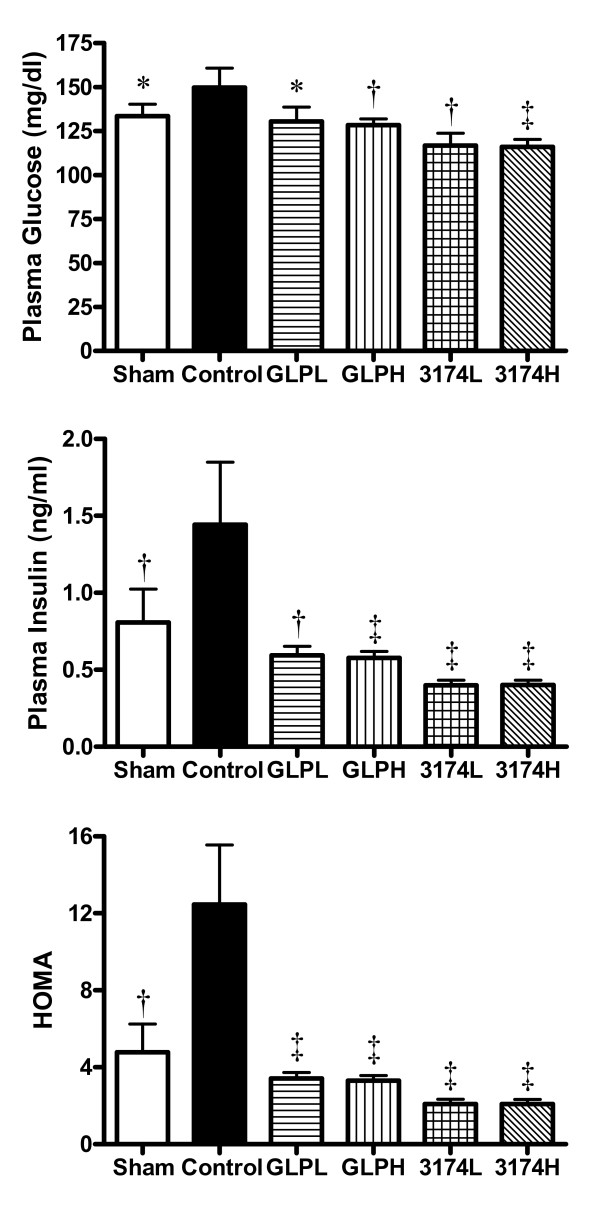
**Effects of GLP-1 and AC3174 on fasting glucose, insulin levels and HOMA 13 weeks post-MI**. * p < 0.05, † p < 0.01, ‡ p < 0.001 versus vehicle control group. Results are mean + SEM.

Post-MI vehicle control rats showed evidence of CHF, including the development of LV dilatation, and LV systolic and diastolic dysfunction (Table 1; Figure 1). Echocardiographic studies showed progressive differences in LV geometry between vehicle control (infarcted) and sham-operated rats. Post-MI rats had significant LV dilatation 2 weeks after ligation. During the following 11 weeks, there was continued LV chamber enlargement in the vehicle control group. The prominent increase in cavity dimensions in the infarcted hearts resulted in a significant decrease in relative wall thickness (anterior wall/posterior wall thickness) compared with vehicle control rats [31]. Hemodynamic abnormalities were characteristic of rats with CHF. Specifically, vehicle control rats showed significantly depressed LV ± dp/dt and elevated LVEDP (Table 2), while cardiac output/body weight was decreased by 19% (p <0.01) compared with sham-operated rats at 13 weeks post-MI. Control rats at 13 weeks post-MI exhibited hyperglycemia and insulin resistance compared with vehicle sham animals (Figure 2). Thus, the post-MI rats we studied represented a homogenous group with characteristics predisposing them to pathologic LV remodeling and CHF.

**Table 1:** Echocardiographic data in post-MI rats.

	Timepoint	Sham Operated (N = 10)	Vehicle Control (N = 13)	GLPL (N = 11)	GLPH (N = 11)	3174L (N = 11)	3174H (N = 7)
**LVEDD (cm)**	**Baseline**	0.63 ± 0.01	0.93 ± 0.02*	0.89 ± 0.01*	0.88 ± 0.02*	0.86 ± 0.02*	0.85 ± 0.04*
	
	**End of Study**	0.82 ± 0.01†	1.16 ± 0.02	1.04 ± 0.03†	1.03 ± 0.03†	1.07 ± 0.03†	0.98 ± 0.05†

**LVESD (cm)**	**Baseline**	0.34 ± 0.01	0.77 ± 0.02*	0.73 ± 0.02*	0.71 ± 0.02*	0.70 ± 0.03*	0.70 ± 0.03*
	
	**End of Study**	0.05 ± 0.01†	0.99 ± 0.03	0.85 ± 0.03†	0.82 ± 0.04†	0.89 ± 0.04	0.73 ± 0.06†

**FS (%)**	**Baseline**	46 ± 2	17 ± 1*	18 ± 1*	19 ± 1*	18 ± 2*	18 ± 1*
	
	**End of Study**	39 ± 1†	16 ± 1	20 ± 2	21 ± 2†	18 ± 1†	26 ± 3†

**LVEDV (ml)**	**Baseline**	0.26 ± 0.02	0.59 ± 0.03*	0.52 ± 0.03*	0.53 ± 0.03*	0.56 ± 0.04*	0.52 ± 0.05*
	
	**End of Study**	0.51 ± 0.02†	0.99 ± 0.05	0.83 ± 0.05†	0.72 ± 0.04†	0.77 ± 0.02†	0.73 ± 0.06†

**LVESV (ml)**	**Baseline**	0.08 ± 0.01	0.37 ± 0.03*	0.32 ± 0.02*	0.33 ± 0.03*	0.34 ± 0.03*	0.33 ± 0.04*
	
	**End of Study**	0.17 ± 0.01†	0.69 ± 0.04	0.52 ± 0.05†	0.42 ± 0.04†	0.47 ± 0.03†	0.44 ± 0.07†

**LVEF (%)**	**Baseline**	69 ± 2	37 ± 2*	39 ± 1*	39 ± 2*	39 ± 3*	38 ± 3*
	
	**End of Study**	66 ± 2†	31 ± 2	38 ± 3†	43 ± 3†	39 ± 3†	41 ± 5†

**E Wave (cm/s)**	**End of Study**	0.89 ± 0.04	1.05 ± 0.50	0.93 ± 0.07	0.89 ± 0.05	1.02 ± 0.06	0.95 ± 0.04

**A Wave (cm/s)**	**End of Study**	0.48 ± 0.06†	0.36 ± 0.06	0.42 ± 0.04†	0.43 ± 0.04†	0.45 ± 0.05†	0.44 ± 0.05†

**E/A Ratio**	**Baseline**	1.6 ± 0.1	3.4 ± 0.5*	4.4 ± 0.8*	3.9 ± 0.6*	3.9 ± 0.8*	4.0 ± 0.4*
	
	**End of Study**	2.0 ± 0.2†	4.5 ± 0.8	2.5 ± 0.4†	2.5 ± 0.4†	2.9 ± 0.6†	2.5 ± 0.5†

**E Wave Deceleration (m/s^2^)**	**Baseline**	13.8 ± 0.6	18.5 ± 1.2*	19.2 ± 1.8*	17.7 ± 0.8*	17.9 ± 1.4*	18.0 ± 0.6*
	
	**End of Study**	16 ± 1†	25 ± 3	18 ± 3†	16 ± 2†	19 ± 3	17 ± 2†

**LV Wall Thinning Ratio**	**Baseline**	1.06 ± 0.02	0.87 ± 0.02*	0.89 ± 0.03*	0.89 ± 0.02*	0.89 ± 0.03*	0.88 ± 0.03*
	
	**End of Study**	1.08 ± 0.03†	0.76 ± 0.02	0.84 ± 0.04	0.88 ± 0.04†	0.79 ± 0.04	0.93 ± 0.06†

**Heart Rate (beats/min)**	**Baseline**	273 ± 5	270 ± 7	266 ± 10	292 ± 10	284 ± 7	261 ± 2
	
	**End of Study**	257 ± 15	243 ± 7	269 ± 8	262 ± 6	246 ± 9	258 ± 7

**CO/Body Wt (ml/min/g)**	**End of Study**	0.21 ± 0.01†	0.17 ± 0.01	0.20 ± 0.01†	0.20 ± 0.01†	0.19 ± 0.01†	0.22 ± 0.01†

The LV wall thinning ratio is the ratio of the anterior wall thickness to the posterior wall thickness. Mean ± SEM. *P < 0.05 versus sham-operated group at baseline. †P < 0.05 versus vehicle-treated group at the end of the study. LV, left ventricular; EDD, end-diastolic dimension; ESD, end-systolic dimension; FS, fractional shortening; EDV, end-diastolic volume; ESV, end-systolic volume; EF, ejection fraction; E, early filling wave; A, late filling wave; E/A ratio, Doppler ratio of early to late transmitral flow velocity; Wt, weight; CO, cardiac output.

**Table 2:** Hemodynamic changes at the end of the study.

	Sham Operated (N = 10)	Vehicle Control (N = 12)	GLPL (N = 11)	GLPH (N = 12)	3174L (N = 13)	3174H (N = 7)
**Heart Rate (beats/min)**	252 ± 13	268 ± 21	294 ± 20	295 ± 13	256 ± 16	312 ± 15

**Mean BP (mmHg)**	83 ± 5	80 ± 8	87 ± 7	84 ± 4	72 ± 2	83 ± 4

**LVEDP (mmHg)**	10.6 ± 0.9*	20.9 ± 1.4	12.7 ± 0.01*	12.3 ± 1.6*	14.4 ± 1.4*	13.9 ± 1.7*

**+ dp/dt (mmHg/s)**	5789 ± 430*	4426 ± 541	6157 ± 654*	6044 ± 284*	5234 ± 372	5948 ± 488*

**-dp/dt (mmHg/s)**	5826 ± 655*	3731 ± 564	5450 ± 652*	5582 ± 465*	4424 ± 386	5798 ± 592*

Mean ± SEM. *P < 0.05 versus vehicle-treated group. BP, blood pressure; dp/dt, first derivative of left ventricular pressure over time.

### Echocardiographic characteristics of infarcted rat hearts prior to treatment

In all randomized post-MI rats at baseline, LV systolic function (LVEF, CO/B.W, FS%) was depressed and LV chamber size (LVESD, LVEDD, LVESV and LVEDV) increased compared with sham-operated rats (Figure 1; Table 1). LV diastolic dysfunction was present in post-MI rats, as assessed by pulse-wave Doppler recordings of mitral flow, compared with sham-operated rats. Increased E/A ratio and E wave deceleration rate, characteristics of LV diastolic dysfunction in failing hearts, was observed in all randomized MI rats compared with sham-operated rats. Left atrial volume was also increased (Figure 1). Prior to treatment with a GLP-1R agonist, there were no significant differences in LV or LA chamber size, or systolic or diastolic function among the randomized post-MI rats.

### Hemodynamic and cardiac function after 11 weeks of treatment

#### Control rats

During the 11-week observation period, LV systolic and diastolic function progressively decreased in control rats compared with sham-operated rats (Figure 1, Tables 1, 2). Specifically, in the vehicle control rats at 13 weeks post-MI, LVEF decreased 53% (P < 0.05), FS% decreased 60% (P < 0.05), E/A ratio increased 120% (P < 0.05), and LVEDP increased by 97% (p <0.05) compared with the sham-operated rats.

#### Treated rats

Treatment with GLP-1 and AC3174 significantly improved both diastolic function (E/A ratio, LVEDP, E wave deceleration rate) and systolic function (LVEF, FS%, CO/BW) compared with the vehicle control group over time, although cardiac function did not reach that of sham-operated animals for most parameters (Figure 1, Tables 1, 2). Specifically, LVEDP was significantly reduced by 41% in the GLPH group and 33% in the 3174H group compared with the vehicle control group. Conversely, LVEF was significantly increased by 39% in the GLPH group and 33% in the 3174H group. In the GLPH and 3174H groups, LV contractility (± dp/dt) was also significantly improved (P < 0.05), while HR and BP were not affected.

### Cardiac geometry

#### Control rats

LV chamber size in the vehicle control group increased progressively compared with sham-operated rats during the 11-week observation period, while LV wall thickness ratio was significantly reduced (P < 0.05, Figure 1, Tables 1 and 2). LVEDD was significantly increased by 42%, LVESD was significantly increased by 97%, LVEDV was significantly increased by 95%, and LVESV were significantly increased by 300% in vehicle control post-MI rats 13 weeks post-MI. In vehicle control rats, LV wall thickness ratio was reduced by 30% and LA volume was increased by 79% compared with sham-operated rats 13 weeks post MI.

#### Treated rats

The progression of LV chamber dilatation and thinning of the LV wall was dose-dependently attenuated in both GLP-1 and AC3174 treatment groups (Figure 1, Tables 1 and 2). Compared with the vehicle control group, LVEDV was significantly reduced by 26% (P < 0.05) and LVESV was significantly reduced by 36% (P < 0.05) in the 3174H group. LVEDV was significantly reduced by 27% (P < 0.05) and LVESV was significantly reduced by 39% (P < 0.05) in the GLPH group. Treatment with GLP-1 or AC3174 dose-dependently attenuated the increase of LA volume observed in the vehicle control rats.

### Insulin sensitivity

#### Control rats

Fasting plasma glucose and insulin levels were significantly higher in the vehicle control group compared with the sham-operated group at 13 weeks post-MI (P < 0.05, Figure 2). As a result, insulin resistance as assessed by HOMA, was 2 fold higher than in the sham-operated group.

#### Treated rats

After 11 weeks of treatment, GLP-1 or AC3174 significantly reduced plasma glucose, insulin and HOMA compared with the vehicle-treated/control group (P < 0.05, Figure 2), indicating that insulin sensitivity was improved in all treated groups.

### Treadmill test, Peak VO_2_, and plasma lactate levels

#### Control rats

Basal VO_2 _was comparable, and peak VO_2 _(PVO_2_) was significantly higher than basal VO_2 _among all groups (P < 0.05, Table 3). Compared with sham-operated rats, running distance was significantly decreased by 63% (P < 0.05) and EC was significantly decreased by 63% (P < 0.05) in vehicle treated post-MI rats with CHF.

**Table 3:** Metabolic response during the treadmill test at the end of the study.

		Sham Operated (N = 10)	Vehicle Control (N = 12)	GLPL (N = 11)	GLPH (N = 12)	3174L (N = 13)	3174H (N = 7)
**Plasma Lactate (mM)**	**Basal**	1.7 ± 0.2*	2.7 ± 0.2	1.9 ± 0.2*	1.9 ± 0.2*	2.0 ± 0.2*	2.1 ± 0.1*
	
	**Peak**	2.5 ± 0.2†	3.1 ± 0.4	2.7 ± 0.3†	3.1 ± 0.6†	3.7 ± 0.5†	3.2 ± 0.5†

**VO_2 _(ml/kg/h)**	**Basal**	949 ± 67	1027 ± 84	1040 ± 110	1117 ± 108	1073 ± 77	872 ± 57
	
	**Peak**	2022 ± 155†	1949 ± 290†	2334 ± 311†	2659 ± 329†	2554 ± 307†	1431 ± 155†#

EC (kg*m)		679 ± 127*	253 ± 52	452 ± 58*	547 ± 56*	535 ± 89*	562 ± 167*

**EC/Peak Lactate Ratio**		290 ± 54*	86 ± 18	200 ± 36*	234 ± 44*	163 ± 31*	203 ± 69*

**EC/Peak VO_2 _Ratio**		38.0 ± 7.2*	14.0 ± 3.2	23.4 ± 4.2	24.8 ± 4.3*	24.4 ± 5.4	40.4 ± 12.2*

**Running Distance (m)**		266 ± 50*	99 ± 21	177 ± 22	212 ± 21*	220 ± 36*	251 ± 82*

Mean ± SEM. *P < 0.05 versus vehicle-treated group. †P < 0.05 peak versus basal in same group. #P < 0.05 3174L versus 3174H group. VO_2_, oxygen consumption; EC, exercise capacity.

#### Treated rats

Treatment with GLP-1 or AC3174 significantly improved running distance and EC in rats with CHF compared with vehicle control animals (P < 0.05, Table 3). Compared with the vehicle control group, PVO_2 _was not significantly changed in sham-operated and GLP-1 or AC3174 treated groups. As a result, the EC/PVO_2 _ratio was significantly higher in all groups compared with the vehicle control group (P < 0.05). PVO_2 _in the 3174H group was significantly lower than in the 3174L group (P < 0.05). Of note, the EC/PVO_2 _ratio in the 3174H group was 1.9 fold-higher than the control group.

Immediately after exercise, peak plasma lactate level was significantly higher than basal levels in each group (P < 0.05). Interestingly, basal plasma lactate levels in the vehicle control group were significantly higher than in the sham-operated and GLP-1 or AC3174-treated groups. Although peak plasma lactate levels were comparable among all groups, the EC/lactate ratio was significantly higher in sham-operated and GLP-1 or AC3174 treated groups (P < 0.05), indicating a higher efficiency of glucose utilization.

### Survival

Over the 13-week post-MI period, 25% of the vehicle control group (4 out of 16 rats) died with signs of CHF (e.g., respiratory distress, and general fatigue: defined as a fast respiratory rate and a reluctance to move.). Eight percent of the GLPL group (1 out of 12 rats) died with signs of CHF. There were no deaths in the sham-operated, GLPH, 3174L, or 3174H groups. As shown in Figure 1F, the Kaplan-Meier survival curves indicate increased mortality for the vehicle control MI group compared with the other treatment groups. Trend tests for GLP-1 (p = 0.05) and AC3174 (p < 0.05) showed marginally significant dose responses. There was no significant difference between the GLP-1 and AC3174 treatment groups. The death rate for all rats treated with a GLP-1R agonist was 2% (1 death out of 44 rats).

### Body composition, heart, lung weight and infarct size

Progressive body weight loss was observed in the AC3174-treated groups during the 11-week treatment period. Weight loss was associated with a dose-dependent reduction in fat mass accompanied by preservation of lean body mass (Table 4). In the 3174H group, body weight decreased 17%, fat mass decreased 45% and fluid mass decreased 11% compared with the vehicle control group. By contrast, there were no significant changes in body weight and composition in the GLP-1 treatment groups over the same time period. LV, RV, and lung weights were generally significantly lower in sham, GLP-1 and AC3174 groups compared with the vehicle control group (P < 0.05). Because body weight also decreased in the GLP-1 and AC3174 groups, the ratios of LV, RV, and lung to body weight did not reach significance for all doses of GLP-1 and AC3174, although lung weight was reduced at the higher doses of both agents. There was no significant change in infarct size among any of the groups.

**Table 4:** Whole body and selected organ composition at the end of the study.

	Sham Operated (N = 10)	Vehicle Control (N = 12)	GLPL (N = 11)	GLPH (N = 12)	3174L (N = 13)	3174H (N = 7)
**Body Wt (g)**	511 ± 16	522 ± 17	523 ± 14	518 ± 12	493 ± 10	435 ± 15*

**Baseline Body Wt (g)**	257 ± 7	272 ± 8	270 ± 7	276 ± 10	258 ± 5	258 ± 5

**Fat Mass (g)**	58 ± 6	47 ± 6	45 ± 6	51 ± 9	33 ± 3*	26 ± 5*

**Lean Mass (g)**	35 ± 1	38 ± 1	37 ± 2	35 ± 2	38 ± 2	34 ± 1

**Fluid Mass (g)**	323 ± 12	343 ± 9	329 ± 14	331 ± 15	332 ± 11	305 ± 9*

**LV (g)**	1.07 ± 0.06*	1.36 ± 0.10	1.26 ± 0.05	1.13 ± 0.03*	1.13 ± 0.05*	1.06 ± 0.04*

**RV (g)**	0.22 ± 0.01*	0.28 ± 0.02	0.22 ± 0.01*	0.23 ± 0.01*	0.24 ± 0.01*	0.20 ± 0.02*

**Lung (g)**	1.53 ± 0.05*	2.45 ± 0.05	1.94 ± 0.35	1.66 ± 0.13*	1.48 ± 0.03*	1.51 ± 0.07*

**LV Wt/Body Wt (%)**	0.21 ± 0.01*	0.26 ± 0.01	0.24 ± 0.01	0.22 ± 0.01*	0.23 ± 0.01*	0.24 ± 0.01

**RV Wt/Body Wt (%)**	0.04 ± 0.002*	0.05 ± 0.01	0.04 ± 0.002*	0.04 ± 0.002*	0.05 ± 0.002	0.05 ± 0.002

**Lung Wt/Body Wt (%)**	0.30 ± 0.01*	0.48 ± 0.10	0.37 ± 0.07	0.32 ± 0.02*	0.30 ± 0.01*	0.33 ± 0.01*

**Infarct Size (%)**	0 ± 0	33 ± 4	30 ± 2	29 ± 2	31 ± 3	31 ± 6

Mean ± SEM. *P < 0.05 versus vehicle-treated group. Wt, weight; LV, left ventricle; RV, right ventricle.

### Effects of GLP-1 or AC3174 on myocardial GLUT4 and GLUT1 translocation

**Figure 3:**
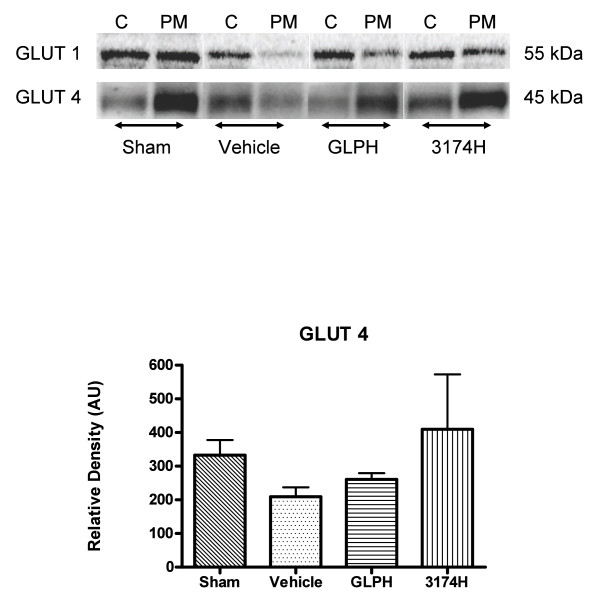
**Effects of GLP-1 or AC3174 treatment on GLUT1 and GLUT4 translocation**. MI rat hearts were obtained from the sham, vehicle control, GLPH and 3174H treatment groups. (A) Representative Western blots of GLUT1 and GLUT4 in plasma membrane and cytoplasm. (B) Quantitative analysis of GLUT4 proteins in plasma membrane. No significant changes with treatment compared to vehicle control were observed. Results are mean + SEM. N = 3 per group.

Figure 3A shows a representative immunoblot of the relative GLUT1 or GLUT4 distribution between the PM and Cyt fractions in sham, vehicle, GLP1 H or 3174H treated MI rats. Antibody binding specifically detected GLUT1 and GLUT4 protein of average molecular mass ~50 kDa and ~45 kDa, respectively, matching the previously described molecular mass of these proteins [37]. Compared with the vehicle control group, treatment with GLP-1 or AC3174 did not change the relative GLUT4 content in the plasma membrane (60.0 ± 4.4% and 61.4 ± 5.5% vs 55.3 ± 4.9%, respectively; Figure 3B). Relative GLUT1 content on the plasma membrane was also unchanged by GLP-1 or AC3174 treatment (37.7 ± 2.9% or 34.8 ± 5.1% vs 41.8 ± 2.5%). These results suggest that GLP-1 or AC3174 do not significantly affect myocardial GLUT1 and GLUT4 translocation under the present experimental conditions.

### Effects of GLP-1 or AC3174 on cardiac protein expression

**Figure 4:**
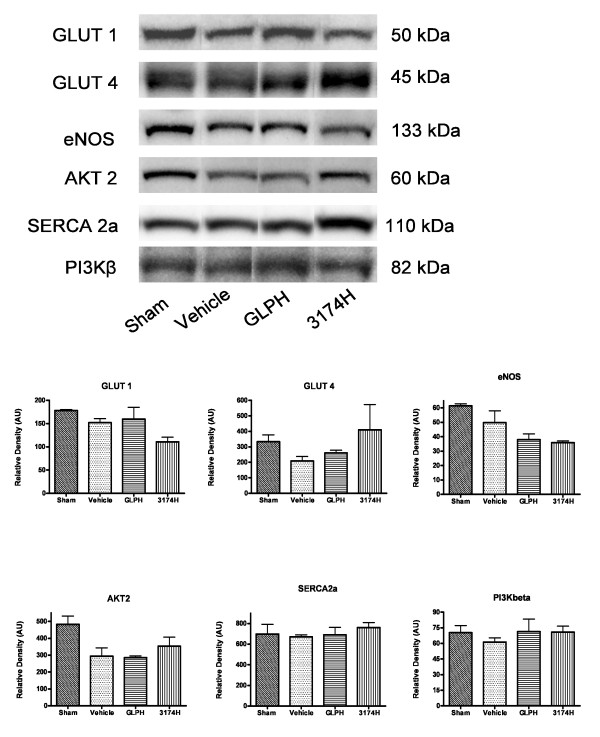
**Effects of GLP-1 and AC3174 on expression of selected cardiac proteins**. MI rat hearts were obtained from the sham, vehicle control, GLPH and 3174H treatment groups. (A) Representative Western blots. (B) Quantitative analysis of cardiac protein expression showing no significant change with treatment compared to vehicle control. Results are mean + SEM. N = 3 per group.

Immunoblot analysis of cardiac tissue did not demonstrate any significant changes in expression of GLUT4, AKT2, SERCA2a or PI3Kbeta with GLP-1 or AC3174 treatment (Figure 4), although trends towards decreased GLUT1 and eNOS expression were noted.

## Discussion

### Overview

The present study demonstrates that 11 weeks of GLP-1 or AC3174 infusion to post-MI rats developing CHF can significantly improve multiple clinically-relevant parameters of cardiac function in a model of moderate, stable, compensated heart failure. In comparison with vehicle-treated rats, improvements were observed in LVEF, fractional shortening, transmitral flow ratio, E-wave velocity, cardiac wall-thinning ratio, LVEDP, dP/dT, and cardiac output. Measured parameters of cardiac morphology were also improved by GLP-1 or AC3174 infusion, including LV end systolic and end diastolic diameter, and left atrial volume. In contrast, heart rate and mean blood pressure in GLP-1R agonist-treated animals were not different from those of vehicle control animals. Left ventricular infarct size was not affected by GLP-1 or AC3174, and no differences in body weight-adjusted measures of heart weight were observed. However, unadjusted LV and RV weights were decreased. Furthermore, a statistically significant improvement in survival was observed with GLP-1 and AC3174 treatments at both low and high doses.

More detailed analyses of the physiological changes resulting from either GLP-1 or AC3174 infusion indicate that fluid balance, glucose metabolism, and respiratory efficiency improved compared with vehicle control animals with CHF. Differences in fluid balance were evidenced by reductions in lung weight (adjusted for body weight) and fluid mass compared with post-MI vehicle control animals. Improvements in glucose metabolism were evidenced by reductions in plasma glucose, plasma insulin, and insulin resistance. Improved exercise capacity in GLP-1 or AC3174-treated animals was associated with reduced peak oxygen consumption during exercise and lower basal lactate production, reflecting improved respiratory efficiency. Running distances in post-MI rats treated with GLP-1 or AC3174 were not significantly different from that of sham-operated animals and were double that of vehicle control animals with CHF.

### Exploration of Possible Mechanisms

These studies did not identify the molecular mechanisms mediating the GLP-1 and AC3174 changes in physiological function. No differences in translocation of GLUT1 or GLUT4 to the plasma membrane were observed between GLP-1 and AC3174-treated rats and no statistically significant differences in expression of these proteins were identified. This contrasts with the results from chronically infarcted Wistar rat hearts where myocardial GLUT4 protein levels were 28% lower in infarcted hearts than in sham-operated hearts, and insulin-stimulated glucose uptake was 42% lower [38]. No differences in the protein expression of AKT2, SERCA2a, or PI3Kbeta were observed, and the observed trend towards reduction in eNOS expression might be expected to diminish cardiac function rather than improve it. However, studies in normal primary human coronary artery endothelial cells *in vitro *have demonstrated a similar lack of overt exenatide effect on eNOS and AKT2 protein expression on a background of enhanced activation of both proteins [39]. Indeed, endothelial cells had a substantial proliferative response to exenatide treatment and this response was mediated by activation of both the AKT2/eNOS and the PKA/PI3K signal transduction pathways. Furthermore, activation of the GLP-1 receptor was required upstream for stimulation of these pathways. Along the same lines of inquiry, treatment of diabetic post-MI mice with the DPP-4 inhibitor sitagliptin, which augments concentrations of endogenous full-length GLP-1, reduced mortality and improved cardiac function [40].

### Role of Glucose Metabolism in CVD

This study demonstrated the development of insulin resistance and hyperglycemia in a MI-induced rat model of CHF, supporting the similarity of this model system with human CHF. Improvements in whole-body insulin sensitivity and glycemic control are closely associated with attenuation of cardiac insulin resistance and appear to protect the heart in both patients and animals with coronary heart disease [14,41]. Several animal studies have shown that increasing glucose utilization not only improves cardiac function, but also attenuates cardiac remodeling during CHF [14,35,42]. Further, a role of GLP-1 in cardioprotection is supported by the cardiac phenotype of GLP-1R knockout mice where resting heart rate is reduced, LV wall thickness is increased, and LVEDP is elevated compare with wild-type mice [43]. Although baseline hemodynamics are normal, after the administration of insulin or epinephrine LV contractility and diastolic function also show impairment.

The close relationship between the metabolic syndrome and cardiovascular diseases, including CHF, is well established [4,11-13,20,25,38]. In previous studies, exenatide progressively reduced body weight in obese animals and humans, and increased insulin sensitivity in obese animals [6,44-47]. In the present study, the combined actions of the exenatide analog AC3174 to reduce body weight, fat mass, insulin resistance, cardiac remodeling, and improve glycemic control and cardiac function suggest the overall improvement in metabolic status observed with AC3174 treatment may contribute to its cardioprotective mechanisms. Further evidence was provided by the decreased rates of mortality in AC3174-treated MI rats compared with vehicle control rats. In patients with CHF and diabetes, but not in normoglycemic patients with CHF, a 5 week infusion of GLP-1 significantly reduced plasma glucose levels [23]. However, cardiac function in both groups of patients was significantly and comparably improved by GLP-1. These results suggest that GLP-1 effects that are independent of whole body metabolic improvements contribute relatively more to GLP-1's cardioprotective effects, perhaps via direct myocardial actions.

In a global ischemia model in isolated rat hearts, GLP-1 treatment post-MI exhibited only a small tendency to increase mechanical (inotropic) performance [48]. Rather, GLP-1's primary mechanism of action was cardioprotective in nature (39% reduction in infarct size) and mediated through the GLP-1 receptor. In isolated mouse hearts, GLP-1 increased functional recovery and cardiomyocyte viability after ischemia-reperfusion injury [49]. In models of MI (ischemia with or without reperfusion) and heart failure, treatment with GLP-1 or exenatide treatment has generally been associated with improvements in post-ischemia cardiac function or infarct size. The most striking results were observed in studies with longer follow-up times. For example, in pigs treated for 2 days, exenatide reduced infarct area 33%, prevented deterioration of systolic and diastolic cardiac function, and decreased myocardial stiffness 54% when assessed on the third day after treatment initiation [19]. At the molecular level, AKT activation increased in concert with increased expression of anti-apoptotic BCL-2 and decreased expression of pro-apoptotic caspase 3. In a second example, 7 days of pre-MI treatment with the GLP-1R agonist liraglutide reduced mouse cardiac infarct size, while improving cardiac output and survival [9]. Four weeks post-MI, measures of systolic function (cardiac output, stroke volume) and mitral flow velocities (E/A ratio) were significantly improved compared with sham-operated mice, combined with reduced LV dilatation. Furthermore, all these effects were independent of liraglutide-induced weight loss. Ex vivo, liraglutide prevented ischemia-reperfusion injury in isolated, perfused mouse hearts and reduced apoptosis in neonatal mouse cardiomyocytes. In normal healthy mice (without MI), liraglutide increased AKT activation, a response that was absent in GLP-1R knockout mice. In the one study examining human patients with acute MI, 72-hours of GLP-1 infusion added to standard therapy was associated with significantly improved LVEF (29% to 39% compared with no change in the control group) and contractile function (-21% in regional wall motion score index versus no change in the control group) measured 6 to 12 hours after infusion [22]. Moreover, in pigs and dogs GLP-1 improved myocardial glucose-uptake and metabolism [50,51].

### Clinical Evidence

The ability of exenatide to reduce blood pressure in humans may contribute to the peptide's potential to play a cardioprotective role. In an open-label, 82-week study in patients with type 2 diabetes, exenatide reduced mean diastolic blood pressure and improved lipid profiles [44]. In a 24-week, clinical trial in patients with type 2 diabetes, exenatide reduced mean systolic and diastolic blood pressure in contrast to non-significant changes in the placebo arm [47]. The blood pressure effects of exenatide treatment lasting at least 6 months was also examined in pooled data from 6 trials including 2,171 subjects [52]. Exenatide was associated with significantly decreased systolic BP compared with placebo or insulin in patients with elevated BP at baseline, with the greatest effects observed in subjects with baseline systolic BP ≥130 mmHg.

In another study, 12 weeks of exenatide treatment in patients with type 2 diabetes was associated with a trend towards lower 24-hr, daytime, and nighttime systolic blood pressure, but had no clinically meaningful effect on heart rate, compared with placebo [53]. Further, using a well-established risk-assessment model, Sullivan et al. [54] projected substantial reductions in cardiovascular death rates and fewer cardiovascular events over 30 years in patients with diabetes treated with the GLP-1R agonist, liraglutide.

Exercise intolerance is a hallmark symptom of CHF regardless of disease etiology, and is closely related to increased insulin resistance [55]. Agents that stimulate glucose oxidation (directly or indirectly) improve exercise capacity in humans [56-58]. In the present study, a reduction in basal plasma lactate and an increase in the ratio of exercise capacity to the lactate peak during exercise was observed with GLP-1 or AC3174 treatment, in parallel with increased insulin sensitivity. These data suggest whole body glucose utilization was improved in all treatment groups. Thus, it is possible that normalization of hyperglycemia and improvement in insulin sensitivity may have contributed to the enhancement of exercise performance, in addition to the benefits of improved cardiac function and remodeling. However, whether or not the improvements in insulin sensitivity associated with chronic GLP-1 or AC3174 treatment directly contributed to the cardioprotective effects of these peptides remains to be determined. Regarding the significantly lower VO_2 _levels observed in the 3174H group, reduced food intake/body weight may have contributed to this result. Previous studies have shown that equivalent doses of exenatide lower food intake in diet induced obese rats [46]. However, the mechanism of action of AC3174 to change VO_2 _is not clear and is likely to be multifactorial.

### Implications of GLP-R Activation for Survival after MI

Survival increased with GLP-1 or AC3174 treatment in the MI-induced CHF rat model. Although attenuation of insulin resistance by GLP-1 or AC3174 may contribute to this benefit, insulin-independent cardiac or extra-cardiac actions such as vasodilatation, renoprotection, and reduction of apoptosis [2,59-61] may have also contributed to the reduction in mortality. Of mention, insulin sensitizers such as peroxisome proliferators-activated receptor γ (PPARγ) activators (e.g., thiazolidinediones) have cardioprotective effects similar to GLP-1. However, PPARγ activators are contraindicated in CHF due to their propensity to increase the incidence of fluid retention and edema in humans [62], and increase mortality in rats with MI-induced CHF [63].

While the mechanisms of the observed cardioprotective effects remain unclear, several likely mechanisms were explored. In a previous study of isolated perfused rat hearts subjected to ischemia and reperfusion, acute treatment with high concentrations of GLP-1 enhanced recovery of cardiac function by improving myocardial glucose uptake and translocation of the glucose transporters, GLUT-1 and GLUT-4, during reperfusion [20]. Although the mechanism of translocation remains elusive, it appears the AKT-2 downstream signal transduction pathway contributes to the translocation of GLUT-4 [64]. In the present study, long term treatment with GLP-1 or AC3174 did not significantly alter myocardial GLUT1 or GLUT4 translocation. These data suggest the observed cardioprotective effects may occur, at least in part, independent of specific cardiac metabolic improvements.

While GLP-1 and the exenatide analogue AC3174 exhibit comparable binding potency at the GLP-1 receptor [7], in this study AC3174 exhibited several distinct pharmacodynamic actions compared with GLP-1. For instance, treatment with AC3174 resulted in significant weight loss mediated by selective loss of body fat. Furthermore, the highest dose of AC3174 tested was associated with a relatively low PVO_2_. This could be due to relatively more potent and sustained inhibitory effects of exenatide on food intake and energy expenditure than observed with GLP-1 [65]. However, the cardioprotective effects of the GLP-1R agonist liraglutide in a mouse MI model were found to be independent of weight reduction [9].

In an isolated rat heart model of MI, administration of GLP-1 during the first 15 minutes post-ischemia reperfusion reduced infarct size through a GLP-1 receptor-mediated pathway, but had no inotropic effects (mechanical performance) [48]. In contrast, administration of the primary GLP-1 metabolite GLP-1(9-36) had no effect on infarct size, but did have a strong negative inotropic effect. Because GLP-1(9-36) has little or no binding affinity for the known GLP-1R, these data suggest the involvement of GLP-1R-independent effects on cardiac function post-MI. A more recent exploration of this hypothesis found that isolated mouse hearts rapidly convert infused GLP-1 to GLP-1(9-36) [66]. After ischemia-reperfusion injury of isolated mouse hearts, administration of GLP-1(9-36) or exenatide improved functional recovery, reduced infarct size, improved cardiomyocyte viability, reduced lactate dehydrogenase release and decreased caspase-3 activation. Counter to expectations, the cardioprotective actions of GLP-1(9-36) were blocked by an antagonist of GLP-1R binding, yet preserved in cardiomyocytes from GLP-1R knockout mice. Overall, these data lend further support to a cardio-sparing signal transduction pathway distinct from that associated with the GLP-1 receptor.

### Limitations

One possible limitation of this study is that standard treatments for MI, e.g. ACE-inhibitors, were not co-administered with GLP-1 or AC3174. However, in a recent publication [60], the ACE inhibitor captopril had additive effects with AC3174 in reducing cardiac left ventricular mass and improving renal morphology in a rat model of hypertension characterized by profound hypertension, cardiac hypertrophy, insulin resistance, renal pathology, and early-onset mortality. AC3174 plus captopril lengthened survival and had anti-hypertensive, insulin-sensitizing, and renoprotective effects. Another possible limitation is that levels of catecholamines, cortisol, glucagon, free fatty acids, renin, and aldosterone were not measured in the MI rat model. Levels of these compounds can increase in heart failure patients and it is possible similar changes may have influenced the physiological responses to AC3174 or GLP-1 in the rat model.

## Conclusions

GLP-1 and the exenatide analog, AC3174, each independently demonstrated cardioprotective effects after long-term treatment in rats with MI-induced CHF, a model of moderate, stable, compensated heart failure. Overall, the cardioprotective benefits of GLP-1 and AC3174 appeared similar, suggesting that in this model, the major GLP-1 metabolite (GLP-1 9-36) is not necessary for mediating these specific improvements. Therefore, based on the findings from the present study and the accumulating body of clinical evidence with exenatide, therapy with GLP-1 receptor agonists may represent a promising approach for the treatment of patients with CHF or cardiovascular disease associated with type 2 diabetes, supporting the need for further research in this field.

## Abbreviations

A: late; CHF: chronic heart failure; CO: cardiac output; Cyt: cytosolic; dp/dt: first derivative of LV pressure over time; DPP-4: dipeptidyl-peptidase-4; E: early; EC: exercise capacity; EDD: end-diastolic dimension; EDP: end-diastolic pressure; EF: ejection fraction; EDV: end-diastolic; ESD: end-systolic dimension; ESV: end-systolic volume; GLP-1: glucagon-like peptide-1; GLUT: glucose transporter; HOMA: homeostasis model assessment; HR: heart rate; LA: left atrial or left atrium; LV: left ventricular or left ventricle; MI: myocardial infarction; PM: plasma membranes; QMR: quantitative magnetic resonance; SERCA2: sarcoplasmic reticulum Ca^2+ ^ATPase; SP: systolic pressure; SV: stroke volume; VO_2_: oxygen consumption; VTI: velocity time integral.

## Competing interests

All authors were employees and held stock in Amylin Pharmaceuticals, Inc. at the time these experiments were performed.

## Authors' contributions

QL helped conceive of the study, and participated in its design, analysis, interpretation, coordination and helped draft the manuscript. CA participated in the design and coordination of the study. AB, CP, and RF participated in the design and coordination of the study and helped carry out the *in vivo *studies and the *in vitro *assays. cell biology and histological studies. AB helped conceive of the study and participated in its design. DGP helped conceive of the study, participated in its design, analysis, interpretation and helped draft the manuscript. All authors read and approved the final manuscript.
